# Bridging model performance and clinical utility in T cell receptor‐epitope binding prediction

**DOI:** 10.1002/ctm2.70837

**Published:** 2026-09-16

**Authors:** Yuyan Wang, Yanping Lu, Shengbao Suo

**Affiliations:** ^1^ Department of Bioinformatics and Artificial Intelligence Guangzhou National Laboratory Guangzhou China

## 1 INTRODUCTION

T cell receptor (TCR) recognition is dependent on the presentation of peptide antigens as epitopes by major histocompatibility complex (MHC) molecules.^1^ This interaction dictates the capacity of T cells to eliminate infected or malignant cells.^2^, ^3^ In oncology, personalised neoantigen vaccines,^4^ engineered TCR‐T cells,^5^ and antigen‐specific T cell monitoring all depend on identifying which TCRs recognise which epitopes.^6^ However, experimental identification remains slow, costly and sample‐intensive, so a reliable computational model holds significant translational value if it can effectively prioritise candidates to guide downstream experiments. Ultimately, clinicians need help deciding which TCR to engineer, which neoantigen to include and which T cell population to expand, rather than yet another balanced‐benchmark accuracy score. Therefore, a model's clinical utility lies in its effect on real‐world applications, not in prediction performance alone.

**FIGURE 1:**
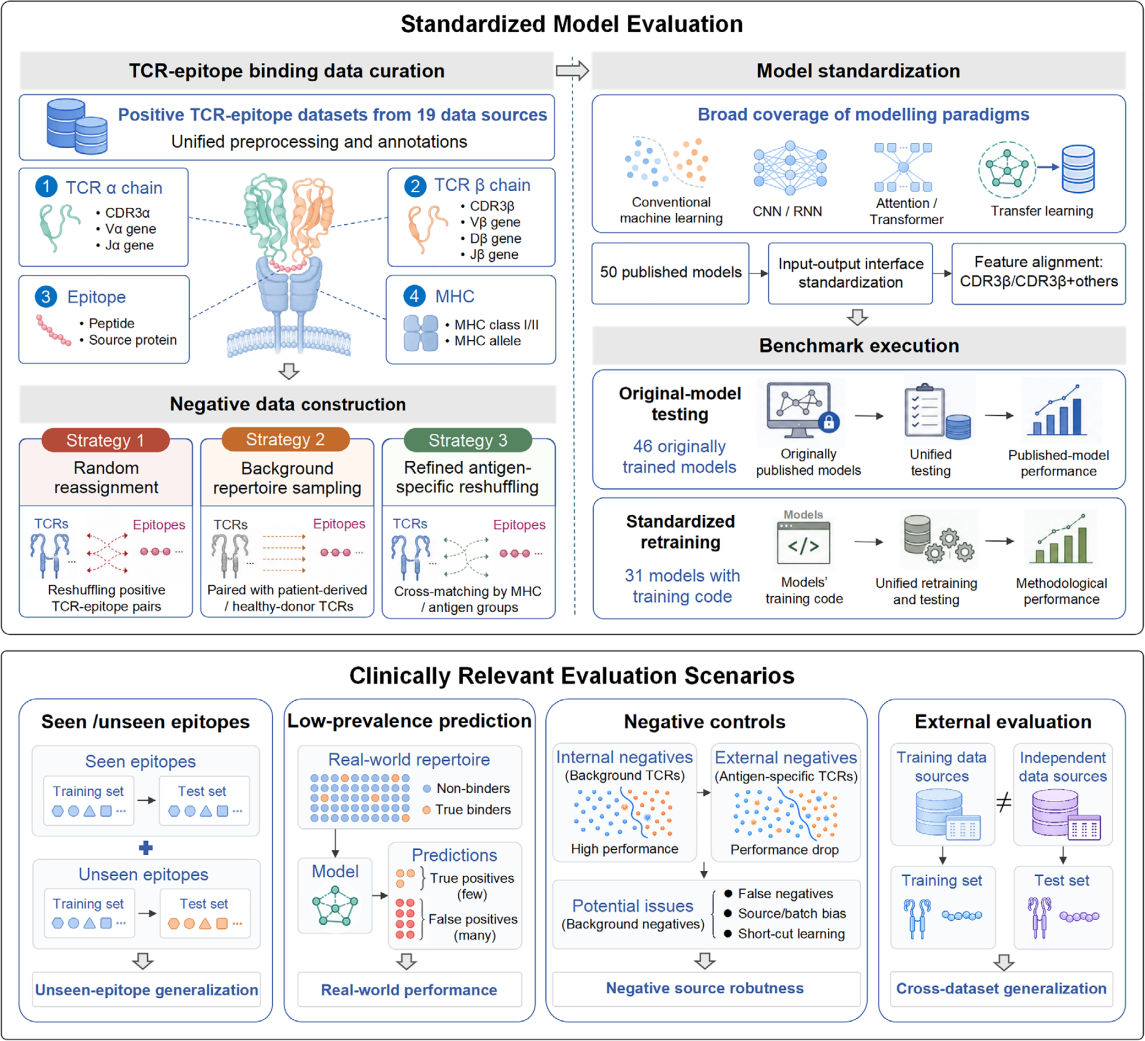
Unified benchmarking framework for TCR‐epitope binding prediction. The framework systematically evaluates 50 published models using large‐scale TCR‐epitope binding datasets through both original‐model testing and standardised retraining under harmonised input‐output settings. Four clinically relevant evaluation scenarios were further incorporated to assess model generalizability, robustness and translational potential in real‐world applications.

To determine this, we conducted a systematic evaluation of 50 published TCR‐epitope binding prediction models. Moving beyond author‐selected metrics, we utilised original implementations where possible and retrained all available models under standardised data partitions and evaluation procedures. We specifically assessed generalisation to epitopes not seen during training, negative‐sample construction, performance when true binders were rare and transferability to independent datasets.^7^ This framework was designed to reflect the way these tools would actually be used in clinical and translational research (Figure 1).

## 2 CORE CLINICAL BARRIERS TO RELIABLE TCR‐EPITOPE PREDICTION

The most consistent finding of our evaluation is that model performance depended heavily on whether an epitope was presented in the training set. While several models could rank candidate TCRs usefully for well‐represented epitopes, their performance collapsed towards random guessing for unseen epitopes. This distinction is far from an edge case. Predicting interactions for personalised neoantigens, newly arising viral variants and less‐studied tumour antigens inherently requires extrapolation beyond the training distribution. Consequently, if a model cannot reliably rank unseen‐epitope interactions, its utility in those contexts should be strictly confined to hypothesis generation rather than confident decision‐making.

Furthermore, the selection of negative controls profoundly shaped apparent performance in ways that are easy to overlook. When non‐binding TCRs are sourced from healthy donors or patient background repertoires, models tend to learn dataset‐specific artefacts, such as batch effects or repertoire characteristics, rather than biological rules of recognition.^8^ While such models may perform well internally, their performance degraded sharply when evaluated on antigen‐specific negative sets. Conversely, biologically constrained negative construction, such as antigen‐specific reshuffling, more directly assesses whether a TCR recognises a particular epitope while mitigating the risk of false negatives. For clinical researchers building a local model from institutional sequencing data, this implies that robust internal performance may reflect technical biases rather than true biological specificity. Thus, negative predictions from such models should be interpreted with particular caution.

Finally, the low prevalence of true binders creates another gap between benchmark metrics and clinical utility. In physiological repertoires, true binders are exceedingly rare, with the vast majority of T cells acting as bystanders. A model exhibiting high sensitivity on a balanced validation set may still yield an unacceptably high false‐positive rate when applied to large‐scale repertoire screening. Therefore, computational models should be used to prioritise a short list for laboratory confirmation, not as standalone diagnostics of antigen specificity. Moreover, independent external validation is equally important. Internal splits and cross‐validation tended to overestimate generalizability, a familiar limitation in clinical prediction modelling. Unless a model has been rigorously tested on genuinely external data, its reported performance metrics should be treated with scepticism.

## 3 CLINICAL IMPLICATIONS OF TCR‐EPITOPE PREDICTION MODELS

These observations prompt three questions for clinical researchers evaluating reported model performance: Was the model tested on epitopes it had never seen? Was it validated on data from an independent source? And does the precision estimate account for the low prevalence of true binders in the intended application? If any answer to any of these is negative, the reported metrics are likely optimistic. Furthermore, while incorporating broader immunogenetic context generally improved predictions, this improvement is not universally consistent. Information such as MHC class and allele, V and J gene usage and paired α and β chains provides critical insights that CDR3β alone cannot capture. Given that many clinical datasets retain only CDR3β data, institutions that intend to build or apply these models should preserve MHC typing and paired‐chain sequencing whenever possible. Additionally, the inherent scarcity of paired αβ TCR data and the input constraints of some models represent practical limitations that further reduce the number of usable clinical pairs.^9^

Nevertheless, these limitations do not mean computational prediction is useless. For well‐characterised viral epitopes or highly represented targets, models could reduce the experimental burden required to identify reactive clones and help interrogate large‐scale sequencing datasets for potential antigen‐specific responses. However, for unseen epitopes, rare binders and cross‐MHC prediction, experimental validation remains indispensable. The appropriate workflow entails computational prediction to prioritise candidates, followed by orthogonal confirmation using multimers, activation markers, or cytokine production assays. The computational score should guide which experiments to perform first, not replace the functional readouts.^10^

## 4 FUTURE OUTLOOK

Looking forward, TCR‐epitope prediction should be viewed as a high‐throughput screening tool rather than a definitive diagnostic test. Its primary clinical value lies in prioritising candidate TCRs and epitopes for experimental validation, particularly when patient material is limited. Currently, the principal bottleneck to further advancement might not be algorithmic capability, but rather the scarcity of high‐quality, well‐curated data. Future progress will therefore necessitate the generation of experimentally confirmed TCR‐epitope pairs, comprehensive paired αβ TCR chain sequences and rigorous independent validation on unseen data. Concurrently, structural information from TCR‐epitope complexes would offer a complementary path for innovation. By elucidating the specific residues that mediate contacts between the peptide‐MHC complex and TCR, structural models can facilitate the learning of biologically interpretable rules, thereby enhancing generalisation to novel epitopes. Furthermore, these structural insights are crucial for elucidating cross‐reactivity and assessing off‐target risk, which matter for the safety of engineered TCR therapies. Ultimately, empowered by richer data and structure‐aware models, computational prediction would reduce the burden of functional assays, preserve precious clinical samples and accelerate TCR discovery, vaccine design, immune monitoring and personalised targeting.

## AUTHOR CONTRIBUTIONS

S.S. conceptualized and supervised the project. Y.W., Y.L. and S.S. interpreted and synthesized the clinical and translational implications of the benchmarking findings and developed the perspectives presented in this manuscript. Y.W. prepared the figures. Y.W., Y.L., and S.S. wrote the manuscript. All authors reviewed and approved the final manuscript.

## CONFLICT OF INTEREST STATEMENT

The authors declare no conflict of interest.

## ETHICS STATEMENT

Not applicable. This study did not involve human participants, animals, or the collection of identifiable personal data.
